# Sputter Epitaxy of ZnO Films on Sapphire Substrates Using 3D MgO Buffer Layers

**DOI:** 10.1021/acsomega.6c04254

**Published:** 2026-07-10

**Authors:** Hiroki Otsuyama, Takafumi Yunoue, Katsuyuki Harada, Kohei Shima, Takamasa Usami, Daisuke Nakamura, Kohei Hamaya, Shigefusa F. Chichibu, Naho Itagaki

**Affiliations:** † Graduate School of Electrical Engineering and Information Science, 12923 Kyushu University, 744, Motooka, Nishi-Ku, Fukuoka 819-0395, Japan; ‡ Institute of Multidisciplinary Research for Advanced Materials, 13101 Tohoku University, 2-1-1, Katahira, Aoba-Ku, Sendai 980-8577, Japan; § Spintronics Research Network Division, Institute for Open and Transdisciplinary Research Initiatives, The University of Osaka, 2-1 Yamadaoka, Suita, Osaka 565-0871, Japan; ∥ Center for Spintronics Research Network, Graduate School of Engineering Science, The University of Osaka, 1-3 Machikaneyama-Cho, Toyonaka, Osaka 560-8531, Japan

## Abstract

Ultrathin MgO buffers composed of three-dimensional (3D) islands enable sputter epitaxy of atomically flat ZnO films on sapphire, despite an 18% lattice mismatch. The resulting 500 nm-thick ZnO films exhibit an exceptionally narrow full width at half-maximum (fwhm) of 21 arcsec in the 0002 X-ray rocking curve (XRC) and a root-mean-square roughness of only 0.11 nm. These improvements are attributed to the 3D MgO islands, which relax the strain at the MgO/sapphire interface (∼17% mismatch), together with structural features more consistent with the metastable wurtzite phase than with the rocksalt one, thereby providing a favorable template with only 1–2% mismatch with ZnO. In contrast, when the MgO buffers convert to the thermodynamically stable rocksalt phase, the ZnO crystalline quality degrades drastically, with the XRC-fwhm exceeding 1000 arcsec due to the larger mismatch (7%) between the rocksalt MgO and wurtzite ZnO. Furthermore, the clear donor-bound exciton peaks observed in the low-temperature cathodoluminescence confirm the satisfactory optical and structural qualities of the ZnO films. These results demonstrate that 3D wurtzite MgO buffers provide an effective strategy for high-quality epitaxy of ZnO and can be extended to other wurtzite materials on lattice-mismatched substrates.

## Graphical abstract

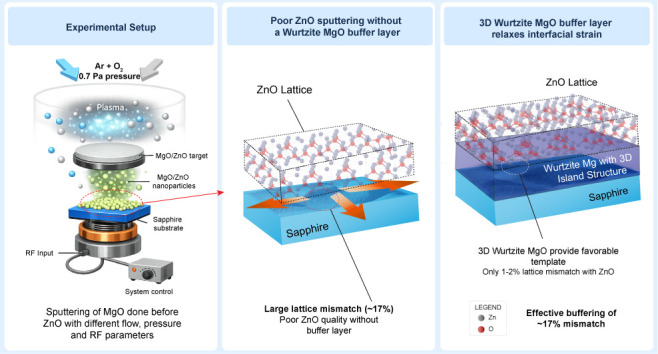

## Introduction

ZnO, a wide-band gap semiconductor, is known for its unique properties including a high exciton binding energy (60 meV),^1^ large piezoelectric coefficient,^2^ strong radiation tolerance,^3^ and the composition from Earth-abundant elements. Owing to these features, ZnO has been regarded as a promising material for a wide range of practical applications,^3^ such as UV-light emitters,^4,5^ UV photodetectors,^6^ transparent conductive oxides,^7,8^ and surface acoustic wave devices.^9,10^ Although research on ZnO for these devices dates back several decades, recent technological advances have brought renewed attention to this material. In addition to their established roles, ZnO-based devices are now being actively explored for resistive and ferroelectric memories,^11–14^ which offer promising applications ranging from conventional data storage to neuromorphic and multilevel computing applications. Owing to its exceptional radiation hardness, ZnO has also attracted increasing attention as a strong candidate for space electronics.^3,15,16^ Furthermore, in the field of photocatalysis, ZnO has garnered considerable interest for solar-driven hydrogen production due to its high redox potential, tunable morphology, and environmental benignity.^17,18^ The broad and growing application portfolio opens up the possibility of designing multifunctional ZnO-based systems that integrate diverse electronic, optoelectronic, and catalytic functions.

Despite their advantages, further development of ZnO-based devices requires advances in the growth of high-quality ZnO films on cost-effective substrates. A major challenge in this regard is the significant lattice mismatch between ZnO and these substrates. For example, sapphire has been widely employed as a substrate for ZnO epitaxy because of its low cost, high crystalline quality, availability in large wafer sizes (up to 12 in.), and a crystal structure that, although formally trigonal, is often treated as hexagonal and thus regarded as compatible with ZnO. However, ZnO films grown on sapphire usually suffer from substantial crystal mosaicity, high dislocation density, poor surface flatness, and numerous grain boundaries, drawbacks primarily caused by the large lattice mismatch of 18% between c-plane sapphire and ZnO.^19–21^

Efforts have been made for improving the quality of ZnO films on sapphire substrates using low-temperature (LT) buffer layers.^19,22,23^ This approach is inspired by the successful high-quality epitaxial growth of GaN on sapphire, despite a large lattice mismatch of approximately 16%.^24,25^ In GaN growth, the LT buffer layer, comprising fine crystallites in an amorphous matrix formed by precise temperature control, provides oriented nucleation sites and lowers the interfacial free energy, enabling lateral growth and coalescence into a larger crystalline film with finite mosaics. While this approach has proven effective for GaN, it appears to have limited effectiveness for ZnO, which readily crystallizes even at room temperature, thereby hindering the formation of the amorphous matrix needed to embed fine crystallites. Meanwhile, although limited in scope, several noteworthy studies have reported interesting results regarding the introduction of an additional MgO interlayer under the LT-ZnO buffer. For example, Kato et al. demonstrated that introducing a thin MgO interlayer before forming an LT-ZnO buffer layer improved the crystal quality of ZnO films.^26,27^ Although MgO is thermodynamically stable in a cubic rocksalt structure, it can also adopt a metastable wurtzite phase with only a 1–2% lattice mismatch relative to ZnO.^28–31^ When deposited on sapphire at thicknesses below 2 nm, MgO tends to form a wurtzite structure; in particular, it exhibits coherent two-dimensional (2D) layer-by-layer growth below 1 nm, whereas it transits to three-dimensional (3D) island growth above this threshold.^32^ The aforementioned improvement in the ZnO crystal quality resulting from the MgO interlayer has been attributed to the presence of this 2D wurtzite-type MgO layer, which provides a structurally favorable template for subsequent LT-ZnO buffer layer growth.^26,27^

Here, we focus on the case after MgO has transitioned to 3D island growth. Reinterpreting the results of the MgO interlayer reported by Kato et al.,^32^ we note that when the MgO film thickness exceeds 1 nm, the initial coherent 2D wurtzite-MgO layer evolves into nanoscale 3D islands while preserving the wurtzite structure up to ∼2 nm. Based on this observation, we hypothesize that MgO layers in the 1–2 nm thickness range, consisting of discrete 3D islands remaining in the wurtzite phase, can act as strain-relieving buffers even without conventional LT buffers. Although the lattice mismatch with ZnO is only 1–2%, as mentioned above, wurtzite MgO still faces a large mismatch with sapphire (∼18%). However, this mismatch is expected to be effectively mitigated because the nanoscale islands, with their small size and correspondingly large surface-to-volume ratios, can relax the elastic strain at their surfaces, thereby reducing the dislocation density within the islands and maintaining good crystal axis alignment both in-plane and out-of-plane.^33,34^ Such buffers therefore provide well-oriented nucleation sites, allowing the subsequent ZnO film to grow in a two-dimensional mode. This proposed mechanism fundamentally differentiates our approach from earlier studies, where wurtzite MgO was employed as a supplementary 2D wetting layer beneath a conventional low-temperature ZnO buffer. By demonstrating that 3D wurtzite MgO can serve as an effective, standalone buffer, this study provides a simplified pathway for the epitaxial growth of a high-quality ZnO.

In this study, we employ MgO alone as the buffer layer and systematically vary its thickness (*d*_MgO_) to investigate its effectiveness in promoting the epitaxial growth of high-quality ZnO films on sapphire substrates. The structural and optical properties of the resulting ZnO films are evaluated using X-ray diffraction (XRD), reflection high-energy electron diffraction (RHEED), atomic force microscopy (AFM), and cathode luminescence (CL). In addition, the effects of both the MgO and ZnO growth temperatures on the crystal quality and surface morphology of the ZnO films are examined. Based on these results, we discuss the potential of MgO to serve as an effective strain-relieving buffer layer for the epitaxial growth of high-quality ZnO films.

## Experimental Section

All films were prepared using radio frequency (RF) magnetron sputtering. First, MgO buffer layers with thickness of *d*_MgO_ = 1–6 nm were grown on c-plane sapphire substrates at 750–850 °C in an Ar/O_2_ atmosphere. The Ar and O_2_ flow rates were 30 and 5 sccm, respectively, at a total pressure of 0.7 Pa. A 2-in.-diameter MgO target (>99.99% purity) was employed, and an RF power of 50 W was supplied to the target. The thicknesses of the MgO layers were evaluated using X-ray reflectometry. Subsequently, ZnO films were deposited on the MgO buffers at 800–900 °C in an Ar/O_2_ atmosphere. The Ar and O_2_ flow rates were 45 and 5 sccm, respectively, at a total pressure of 0.70 Pa. Two 2-in.-diameter ZnO targets (>99.99% purity) were used, and an RF power of 60 W was supplied to each. The thickness of the ZnO films was determined using scanning electron microscopy. No postdeposition annealing was performed.

The crystal quality of the ZnO films was evaluated by XRD using a four-circle texture diffractometer and Cu Kα1 radiation. Because the MgO buffer layers investigated here were only 1–6 nm thick, conventional XRD analysis did not provide sufficient sensitivity for reliable phase identification of the ultrathin MgO itself. Therefore, the crystal structure of the MgO buffer layers was examined by RHEED at an acceleration voltage of −20 kV and a glancing angle of 1°–2°, yielding an electron beam wavelength of 0.0087 nm and an irradiated area several millimeters long along the beam path. The camera length and screen radius were 233 and 85 mm, respectively. The surface morphologies of both the ZnO films and MgO buffers were characterized using tapping-mode AFM. CL spectra were measured at 15 K using a custom-built CL setup.^35,36^ The electron beam was incident at 60° with an acceleration voltage of −3.5 kV and a probe current of 70 μA, corresponding to a beam diameter of approximately 750 μm and a current density of 16 mA·cm^–2^.

All ZnO films fabricated in this study were confirmed to have grown epitaxially on sapphire substrates. Figure 1 shows the *φ*-scans of the asymmetric 101̅1 reflection for ZnO films grown directly on sapphire and MgO buffers of various thicknesses. Here, *φ* represents the azimuthal angle corresponding to the in-plane rotation during the XRD measurements, while “asymmetric” refers to a diffraction geometry in which the scattering vector is inclined relative to the surface normal. For all samples, diffraction peaks appeared every 60°, indicating epitaxial growth with a 6-fold in-plane rotational symmetry of the hexagonal ZnO lattice. The epitaxial relationships were determined to be [0001]_ZnO_ ||[0001]_Al_2_O_3__ and [101̅0]_ZnO_ ||[112̅0]_Al_2_O_3__.

**Figure 1:**
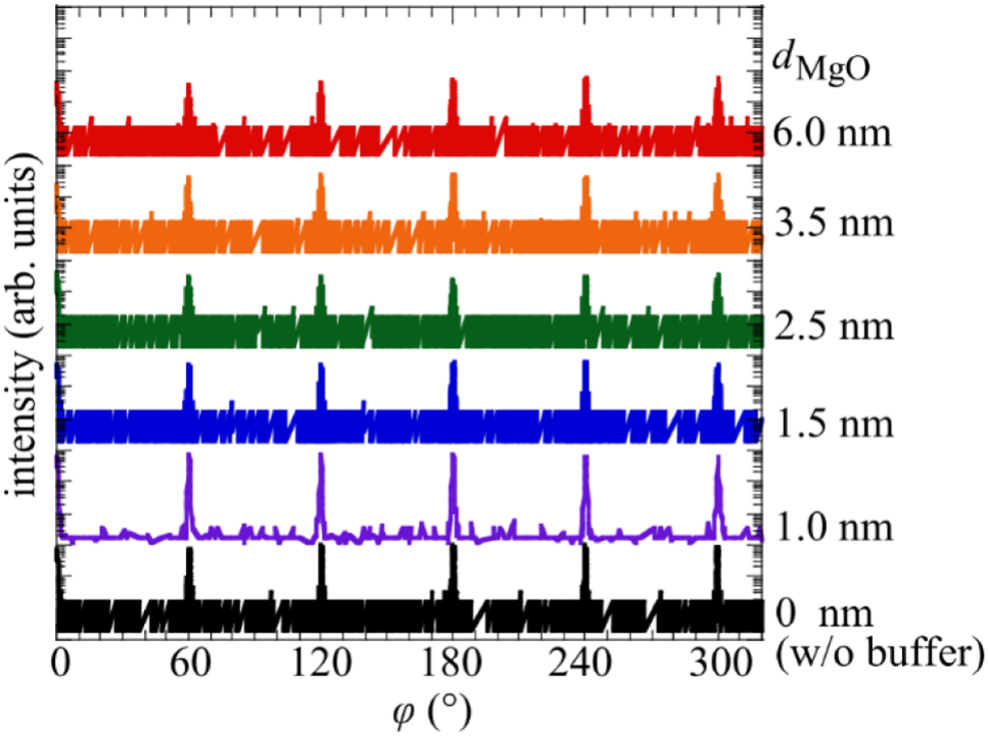
XRD φ-scans of asymmetric 101̅1 reflection for 100 nm-thick ZnO films grown directly on sapphire and on MgO buffers of various thickness.

## Results and Discussion

### Effect of MgO Buffer Layer Thickness on the Crystal Quality of ZnO Films

We found that thin MgO buffer layers (*d*_MgO_ < 2 nm) significantly improved the crystal quality of the subsequently grown ZnO films. Figure 2a shows the X-ray rocking curves (XRC) of the symmetric 0002 reflection from ZnO films grown on MgO buffer layers of various thicknesses, plotted on a logarithmic intensity scale. In all cases, the ZnO film thickness (*d*_ZnO_) was approximately 100 nm, and both MgO and ZnO layers were deposited at 800 °C. For comparison, Figure 2a also includes the XRC of a 100 nm-thick ZnO film grown without a buffer.

**Figure 2:**
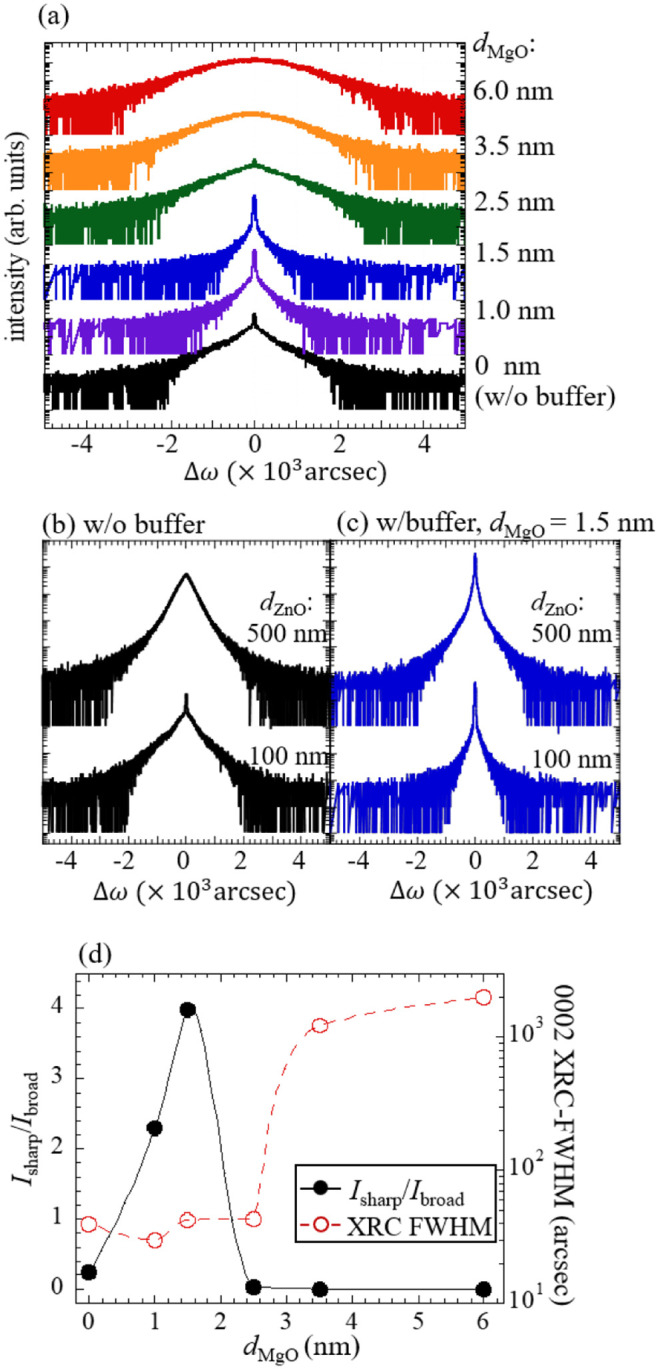
XRC analysis of the symmetric 0002 reflection from the ZnO films. (a) XRCs of 100 nm-thick ZnO films with and without MgO buffers of various thicknesses. (b) XRC of a 500 nm-thick ZnO film grown without a buffer layer. (c) XRC of a 500 nm-thick ZnO film grown on a 1.5 nm-thick MgO buffer. (d) fwhm and integrated intensity ratio of the sharp to broad components (*I*_sharp_/*I*_broad_) of the XRCs as a function of *d*_MgO_.

Overall, introducing an MgO buffer layer thinner than 2 nm enhanced the out-of-plane orientation of the ZnO. In contrast, buffer layers with *d*_MgO_ ≥ 2 nm degraded the crystal quality, resulting in broader XRD peaks.

For the ZnO film grown directly on sapphire, the XRC exhibited two superimposed components: a sharp peak associated with regions of superior out-of-plane alignment and a broad peak originating from poorly aligned regions.^37^ The sharp component, with a full width at half-maximum (fwhm) of 43 arcsec, likely originates from a coherently strained ZnO layer formed only at the film–substrate interface.^37^ Indeed, in thicker ZnO films (*d*_ZnO_ ∼ 500 nm), this sharp peak disappeared and was overshadowed by a broad peak with an fwhm of 281 arcsec, as shown in Figure 2b. This behavior suggests Stranski–Krastanov (SK) growth, in which a 2D coherent ZnO layer initially forms, and after exceeding the critical thickness (where the strain energy surpasses the surface energy gain), the outweighed strain energy leads to 3D growth. This interpretation is consistent with previous reports on ZnO growth on sapphires.^37^ In contrast, a ZnO film grown on a 1.5 nm-thick MgO buffer retained and even strengthened the sharp peak at a film thickness of 500 nm (Figure 2c). Here, the sharp component with a fwhm of 38 arcsec remained dominant, indicating that the buffer relaxed the lattice mismatch and thereby facilitated the growth of ZnO with a superior out-of-plane orientation.

To quantify this effect, we plotted the integrated intensity ratio of the sharp to broad components of the 0002 XRC (*I*_sharp_/*I*_broad_) along with the fwhm values in Figure 2d, where the fwhm was determined directly from the overall peak profile without separating the peaks. For the ZnO film on bare sapphire (*d*_MgO_ = 0 nm), the fwhm was relatively small (40 arcsec), but *I*_sharp_/*I*_broad_ was only ∼0.2, indicating that the well-aligned coherent layer was confined to the interface. In contrast, with MgO buffers of 1–1.5 nm thickness, the ratio increased by nearly 20-fold. We attribute this improvement to the formation of wurtzite MgO layers with thicknesses of 1–2 nm, which exhibit lattice parameters close to ZnO (mismatch 1–2%), and their 3D island morphology effectively relaxes the strain associated with the large mismatch (∼19%) between MgO and sapphire.

To examine this possibility, we first examined the surface morphology of the MgO buffers with thickness of 1–1.5 nm using AFM. Their crystallographic character was then assessed by RHEED, because the ultrathin MgO layer was too thin to provide sufficient diffraction intensity for reliable phase identification by conventional XRD. The AFM images revealed a 3D island morphology, while the RHEED results are more consistent with the metastable wurtzite structure than rocksalt one. Figure 3 shows the AFM images of MgO buffers with different thicknesses. At *d*_MgO_ ∼ 1 nm, atomic steps are observed together with 3D islands on the terraces, whereas at ∼1.5 nm, the step features disappear, and the growth proceeds predominantly in a 3D manner. Remarkably, even under these conditions, the surface retained atomic-scale smoothness, with an RMS roughness of 0.16 nm, suggesting that the 3D islands formed in a highly uniform manner, with their nucleation occurring nearly simultaneously.

**Figure 3:**
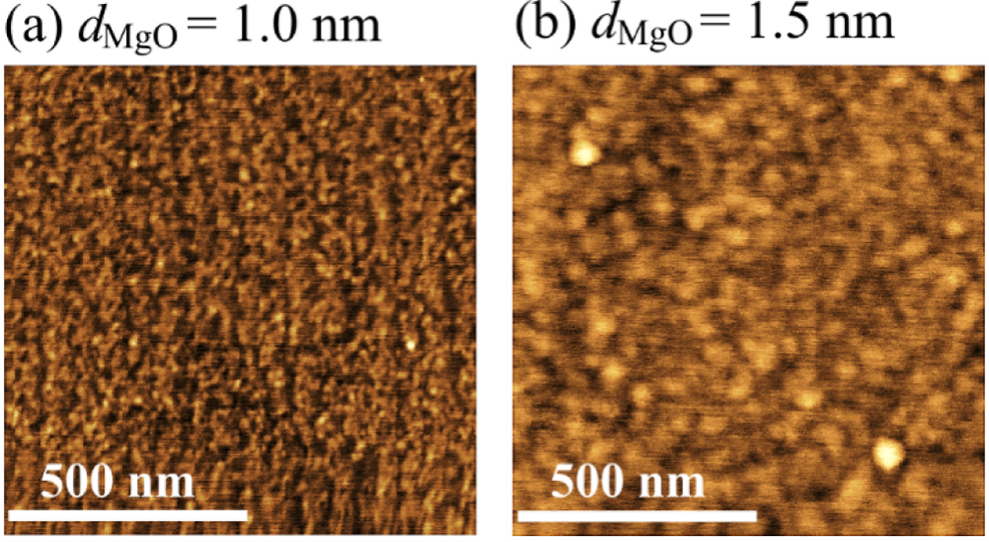
AFM images of MgO buffer layers with thicknesses of (a) *d*_MgO_ = 1.0 nm and (b) *d*_MgO_ = 1.5 nm.

Figure 4 shows the RHEED patterns for the bare sapphire and for a 1.5 nm-thick MgO buffer. The latter exhibited elongated spots superimposed on streaks, indicative of a transition from 2D to 3D growth. From the spot spacings in the RHEED patterns measured with the incident beam aligned along Al_2_O_3_ [101̅0], the interplanar spacings of Al_2_O_3_ (*s*_Al_2_O_3__) and MgO (*s*_MgO_) were calculated as 0.257 and 0.272 nm, respectively (Table 1). The ratio, *s*_MgO_/*s*_Al_2_O_3__, was 1.06, which lies between the fully coherent value of 1 and the literature value of 1.18 for wurtzite (*w*-) MgO (101̅0) relative to Al_2_O_3_ (112̅0) (*s_w‑_*_MgO(101̅0)_/*s*_Al_2_O_3_(112̅0)_).^28^ This intermediate value implies semicoherent growth of *w*-MgO, where the local lattice registry is maintained while part of the misfit is accommodated by relaxation. In contrast, the reported ratio of interplanar spacings for rocksalt (*rs*-) MgO (11̅0) to Al_2_O_3_ (112̅0) (*s_rs_*_‑MgO(11̅0)_/*s*_Al_2_O_3_(112̅0)_) is 1.25 (for example, ref 30), far from the observed value of 1.06, making the interpretation of growth in the rocksalt phase unlikely.^30^ One could hypothetically assume that *rs*-MgO coherently strained to reproduce an observed ratio of 1.06. However, with a lattice mismatch of ∼25% relative to sapphire, which is much larger than the ∼17% mismatch for *w*-MgO, such coherence is unlikely to be sustained at a thickness of 1.5 nm. It is therefore more reasonable to interpret the buffer layers as having a metastable wurtzite structure, consistent with earlier reports.^26,27^

**Figure 4:**
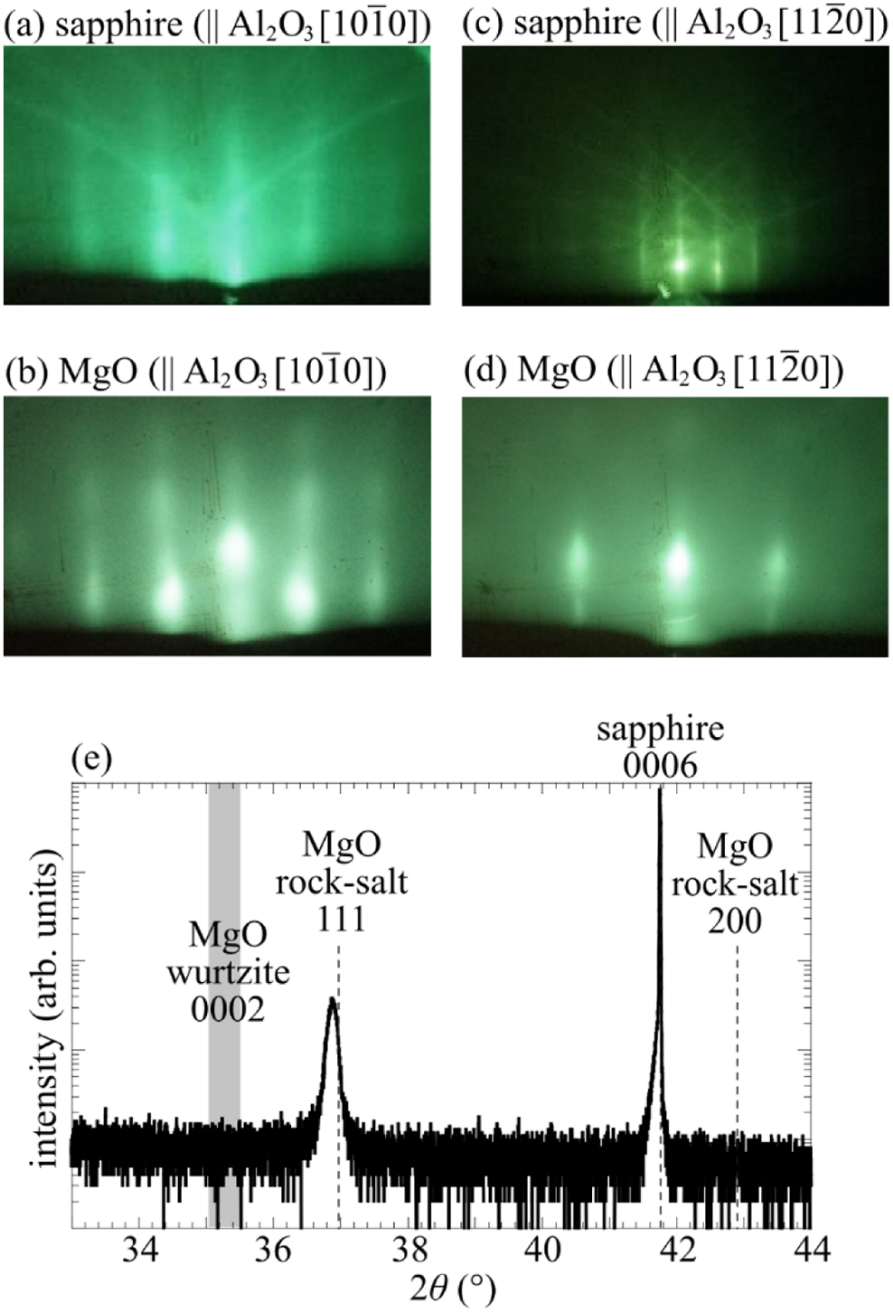
Structural analysis of MgO films. (a–d) RHEED patterns for: (a) bare sapphire and (b) a 1.5 nm-thick MgO buffer (beam direction: Al_2_O_3_ [101̅0]); (c) bare sapphire and (d) 1.5 nm-thick MgO buffer (beam direction: Al_2_O_3_ [112̅0]). (e) XRD θ–2θ scan of a 100 nm-thick MgO film.

**Table 1:** Interplanar Spacings of Sapphire and MgO Buffer Determined from RHEED with Electron Beam Incidence along Specific Crystallographic Directions, Together with the Corresponding Lattice Constants of MgO (*a*_MgO_)­^a^

		interplanar spacings			***a***_**MgO**_ ^b^ (nm)	
		*s*_Al_2_O_3__ (nm)	*s*_MgO_ (nm)	*s*_MgO_/*s*_Al_2_O_3__	wurtzite	rocksalt
RHEED results (beam direction: Al_2_O_3_ [101̅0])		0.257	0.272	1.06	0.291	0.356
Reference values	*w*-MgO (101̅0)^e^	(0.238^c^)	0.282	1.18	0.326	-
	*rs*-MgO(11̅0)^f^	(0.238^c^)	0.298	1.25	-	0.421
RHEED results (beam direction: Al_2_O_3_[112̅0]		0.434	0.156	0.36	0.296	0.363
Reference values	*w*-MgO (112̅0)^e^	(0.412^d^)	0.163	0.39	0.326	-
	*rs*-MgO (112̅)^f^	(0.412^d^)	0.171	0.42	-	0.421

a The reference values for wurtzite (*w*-) and rocksalt (*rs*-) MgO are also listed for comparison.

b The values of *a*_*MgO*_ were calibrated using the sapphire *a*-axis lattice constant.

c 0.238 nm corresponds to the literature value of the interplanar spacings of Al_2_O_3_ (112̅0).

d 0.412 nm corresponds to the literature value of the interplanar spacings of Al_2_O_3_ (101̅0).

e Reference 28.

f Reference 30.

This interpretation is further supported by RHEED patterns measured with the incident beam aligned along Al_2_O_3_ [112̅0], where the ratio of the interplanar spacings for MgO to that of Al_2_O_3_ (*s*_MgO_/*s*_Al_2_O_3__) was 0.36. This value is much closer to the reported 0.39 for *s*_w‑MgO(102̅0)_ */s*_Al_2_O_3_(101̅0)_ than to 0.42 for *s_rs_*_‑MgO(112̅)_/*s*_Al_2_O_3_(101̅0)_.^28^ Moreover, if the layer is assumed to be *rs*-MgO, the derived *a*-axis lattice constant *a*_MgO_ (calculated from the measured interplanar spacings using the sapphire *a*-axis constant as a reference) would be ∼14% smaller than the literature value of 0.421 nm.^30^ Such a large in-plane compression is again unlikely to be sustained under coherent growth. In contrast, assuming *w*-MgO (112̅0) yields *a*_MgO_ ∼0.29 nm, only about 8% smaller than the reported values.^28^ This quantitative agreement, contrasted with the physically unrealistic strain required for the rocksalt phase, further solidifies our earlier conclusion that the MgO buffers are most consistent with a metastable wurtzite structure. The RHEED patterns also revealed that MgO grew on sapphire with a 30° in-plane rotation, corresponding to the epitaxial relationship [101̅0]_MgO_ ||[112̅0]_Al_2_O_3__.

In contrast to the improved crystal quality observed for MgO buffers with thickness of 1–1.5 nm, the degradation in ZnO crystal quality observed for MgO buffers thicker than 2 nm is likely due to a phase transition of MgO into its thermodynamically stable rocksalt structure. Consistent with this interpretation, a much thicker MgO film (*d*_MgO_ ∼ 100 nm) exhibits the rocksalt 111 reflection in XRD, as shown in Figure 4e. Because the MgO buffers fabricated in this study were only a few nanometers thick, reliable phase determination by conventional XRD was difficult. We therefore used the thick-film XRD result only to identify the phase reached in the thick-film limit. This thickness-dependent transition from metastable wurtzite to stable rocksalt MgO is consistent with the findings of Kato et al.^32^ While they combined 2D wurtzite MgO with an LT-ZnO buffer layer,^27^ our results demonstrate that 3D wurtzite MgO islands alone serve as an effective buffer, enabling the epitaxial growth of a high-quality ZnO film on sapphire despite the 18% lattice mismatch.

### Effects of Growth Temperatures of MgO Buffer Layers and ZnO Films on ZnO Crystal Quality

We next investigated how the growth temperatures of the MgO buffer layer (*T*_MgO_) and the ZnO layer (*T*_ZnO_) affect the crystalline quality and surface morphology of the resulting ZnO films. Figure 5 shows the XRCs for ZnO films grown at *T*_ZnO_ of 800 and 900 °C on MgO buffer layers fabricated at *T*_MgO_ ranging from 750 to 850 °C, plotted on a logarithmic intensity scale. The thickness of the MgO buffer was 1.5 nm, and that of the ZnO film was 500 nm. A clear trend was observed, wherein a decrease *in T*_MgO_ led to an improvement in the crystalline quality of the ZnO films. Specifically, the 0002 XRC became sharper with decreasing *T*_MgO_, reaching a minimum fwhm of 37 arcsec at *T*_MgO_ = 750 °C for *T*_ZnO_ = 800 °C.

**Figure 5:**
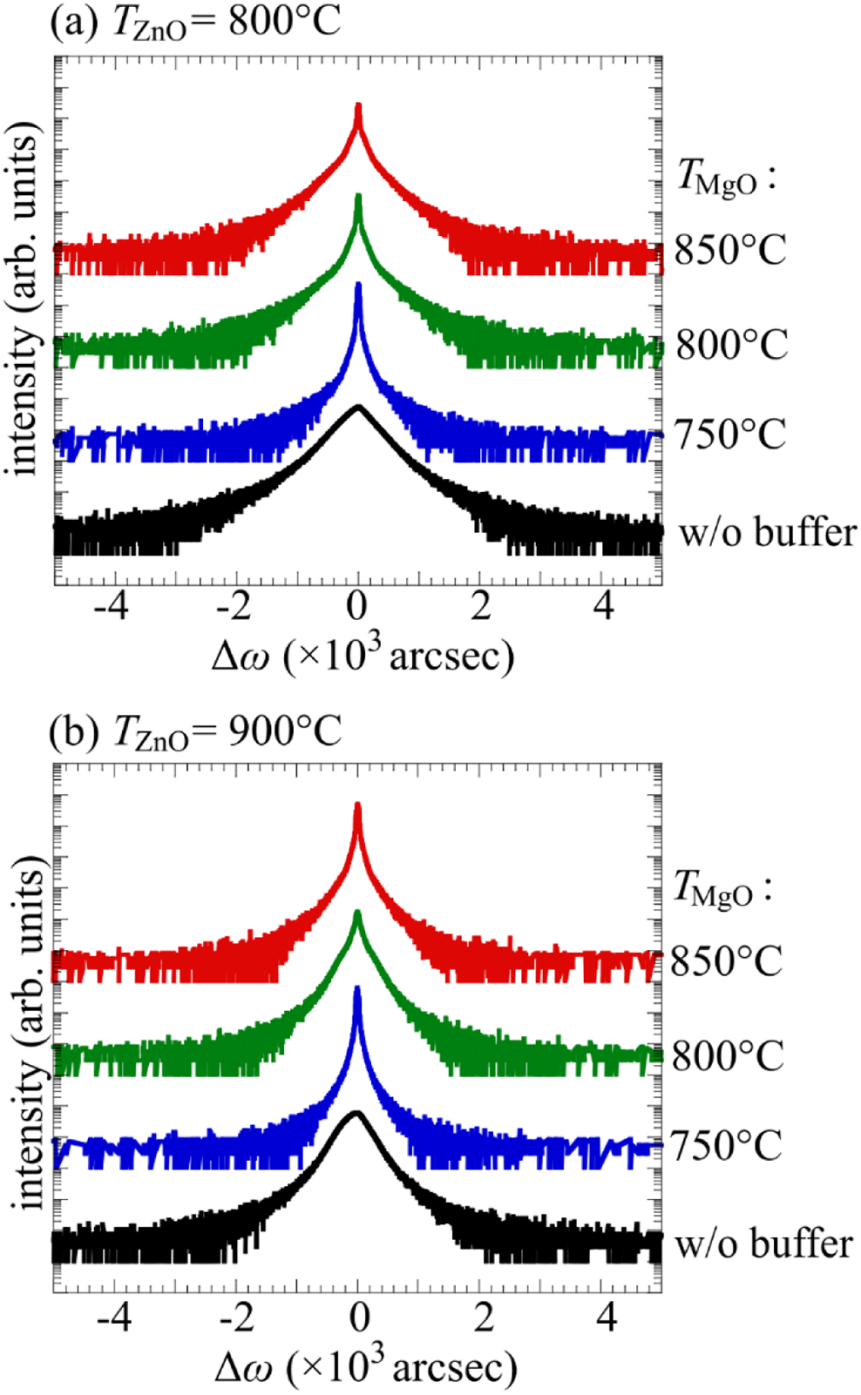
XRCs of the symmetric 0002 reflection from 500 nm-thick ZnO films with and without MgO buffer layers, fabricated at (a) *T*_ZnO_ = 800 °C and (b) *T*_ZnO_ = 900 °C, plotted on a logarithmic intensity scale. The MgO buffer layers were fabricated at *T*_MgO_ = 750–850 °C, and the thickness was 1.5 nm.

Conversely, increasing the *T*_ZnO_ to 900 °C also improved the crystalline quality, with the fwhm narrowing further to as low as 21 arcsec at *T*_ZnO_ = 900 °C. This value is comparable to, or even better than, that achieved for ZnO films grown by molecular beam epitaxy using LT-ZnO buffer layers in combination with 2D MgO layers.^27^ These results suggest that a higher *T*_ZnO_ enhances the 2D growth of ZnO films on the buffer layers, leading to better crystal quality, while a lower *T*_MgO_ likely promotes the formation of smaller 3D islands in the MgO layers owing to somewhat suppressed adatom migration. From the perspective of lattice strain, the formation of such small 3D islands in the buffer layer is crucial for achieving high-quality ZnO films. The island size is affected by the ratio of the incident adatom flux (*F*) to adatom surface diffusivity (*D*). Because the mean surface diffusion length of an adatom before encountering another adatom depends on the *D*/*F* ratio,^38,39^ and *D* follows an Arrhenius-type dependence on temperature, this ratio is influenced by the substrate temperature under a fixed flux of sputtered particles. Thus, a lower *T*_MgO_ reduces the *D*/*F* ratio, resulting in the formation of smaller 3D islands. In addition, a lower *T*_MgO_ may help stabilize the metastable wurtzite phase of MgO.

The surface morphologies of the ZnO films grown using various combinations of *T*_MgO_ and *T*_ZnO_ were further examined using AFM, as shown in Figure 6. The film thicknesses were again ∼1.5 nm for MgO and ∼500 nm for ZnO films. The AFM images revealed that the ZnO films grown on the MgO buffer layers were significantly smoother than those grown directly on sapphire substrates. The presence of atomic steps and terraces on the ZnO surface suggests 2D growth of ZnO, and, when considered together with the previously discussed 3D island morphology of the MgO buffer layers, indicates a transition from 3D to 2D growth. The initial formation of 3D MgO islands is considered to facilitate effective strain relaxation, which subsequently promotes the 2D, layer-by-layer growth of high-quality ZnO on the lattice-mismatched substrate.

**Figure 6:**
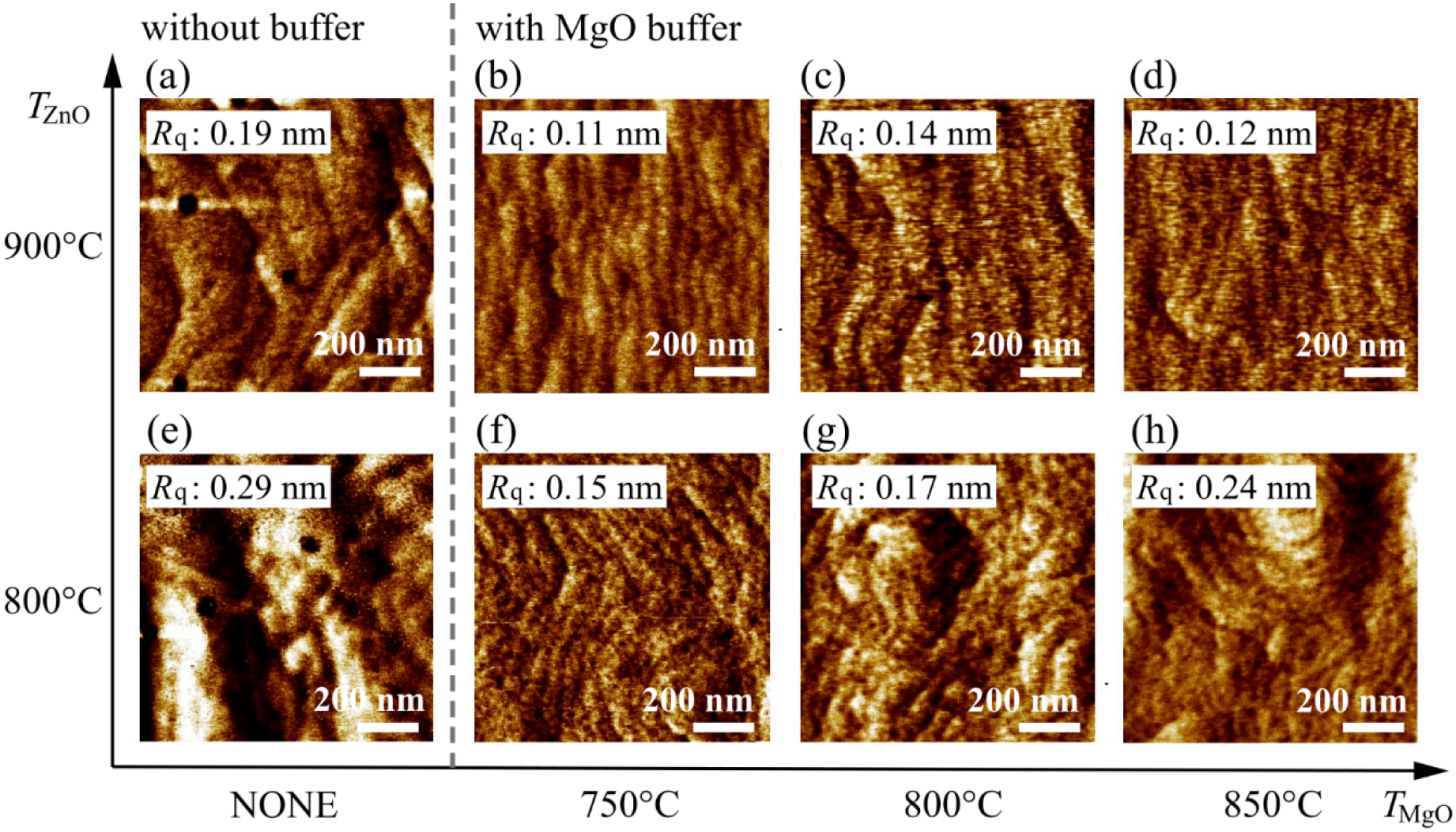
AFM images of 500 nm-thick ZnO films: (a, e) without a buffer layer and (b–d, f–h) with 1.5 nm-thick MgO buffer layers. *T*_ZnO_ = 900 °C for (a–d) and 800 °C for (e–h); *T*_MgO_ = 750 °C for (b, f), 800 °C for (b, f), and 850 °C for (d, h).

The impact of *T*_MgO_ on the surface morphology was also significant. The flattest surface was achieved at *T*_MgO_ = 750 °C. In particular, at *T*_ZnO_ = 800 °C, a clear dependence of surface roughness on *T*_MgO_ was observed, where the RMS roughness decreased markedly from 0.24 to 0.15 nm as *T*_MgO_ decreased from 850 to 750 °C. At *T*_ZnO_ = 900 °C, although low RMS values were maintained across all *T*_MgO_ conditions, the smoothest surface was again achieved at *T*_MgO_ = 750 °C, exhibiting an exceptionally low RMS roughness of 0.11 nm and straight step edges. These findings indicate that combining a lower *T*_MgO_ and a higher *T*_ZnO_ provides suitable conditions for atomically flat, high-quality ZnO film growth, wherein the former promotes 3D island formation in the buffer layer, and the latter promotes 2D, layer-by-layer growth of the ZnO layer.

### Cathodoluminescence Study of ZnO Films on MgO Buffer Layers

CL measurements were carried out on the ZnO film grown at *T*_MgO_ = 750 °C and *T*_ZnO_ = 900 °C, where a high crystalline quality was achieved, as shown in Figure 5. Figure 7a shows the CL spectra of the ZnO film measured at 15 K and room temperature (RT), plotted on a logarithmic intensity scale. A high-resolution spectrum focusing on the near-band-edge (NBE) region at 15 K is presented in Figure 7b. At RT, the NBE emission was observed at 3.31 eV, corresponding to the transition energy of free A excitons (FX_A_). This value is consistent with the ZnO bandgap at RT (∼3.37 eV) minus the exciton binding energy (∼60 meV), the latter of which is generally considered to be temperature independent.^40^ The asymmetric line shape of the NBE emission, featuring a low-energy tail, is likely attributable to longitudinal optical (LO) phonon replicas.^41^ A deep-level emission band centered at 2.3–2.4 eV was also observed, with an intensity approximately one-tenth that of the NBE peak, indicating a relatively low contribution from the midgap recombination centers. These emissions have been commonly attributed to point defects or unintentional impurities.^42,43^ At 15 K, the deep-level emission is further suppressed to less than one-hundredth of the excitonic peak, and the NBE region exhibits fine spectral features corresponding to various excitonic states, as shown in Figure 7b. Prominent emission lines at 3.357 and 3.361 eV are attributed to A-excitons bound to neutral donors (D^0^ X).^44,45^ Based on secondary ion mass spectrometry (SIMS) analysis, which revealed the unintentional incorporation of In and Al at concentrations on the order of 10^16^ and 10^18^ cm^–3^, respectively, these lines were assigned to D^0^ X emissions associated with In and Al, respectively. The emission observed at 3.365 eV is tentatively assigned to B-excitons bound to neutral Al donors, likely originating from the transverse branch of the B-excitons, as suggested in our previous study.^46^ The peak at 3.372 eV is attributed to the recombination of B-excitons bound to neutral Al donors, as the energy difference between this peak and the aforementioned 3.362 eV peak, associated with A-excitons bound to neutral Al donors, is ∼11 meV, closely matching to the reported energy separation between A- and B-free excitons (∼10 meV).^47–49^

**Figure 7:**
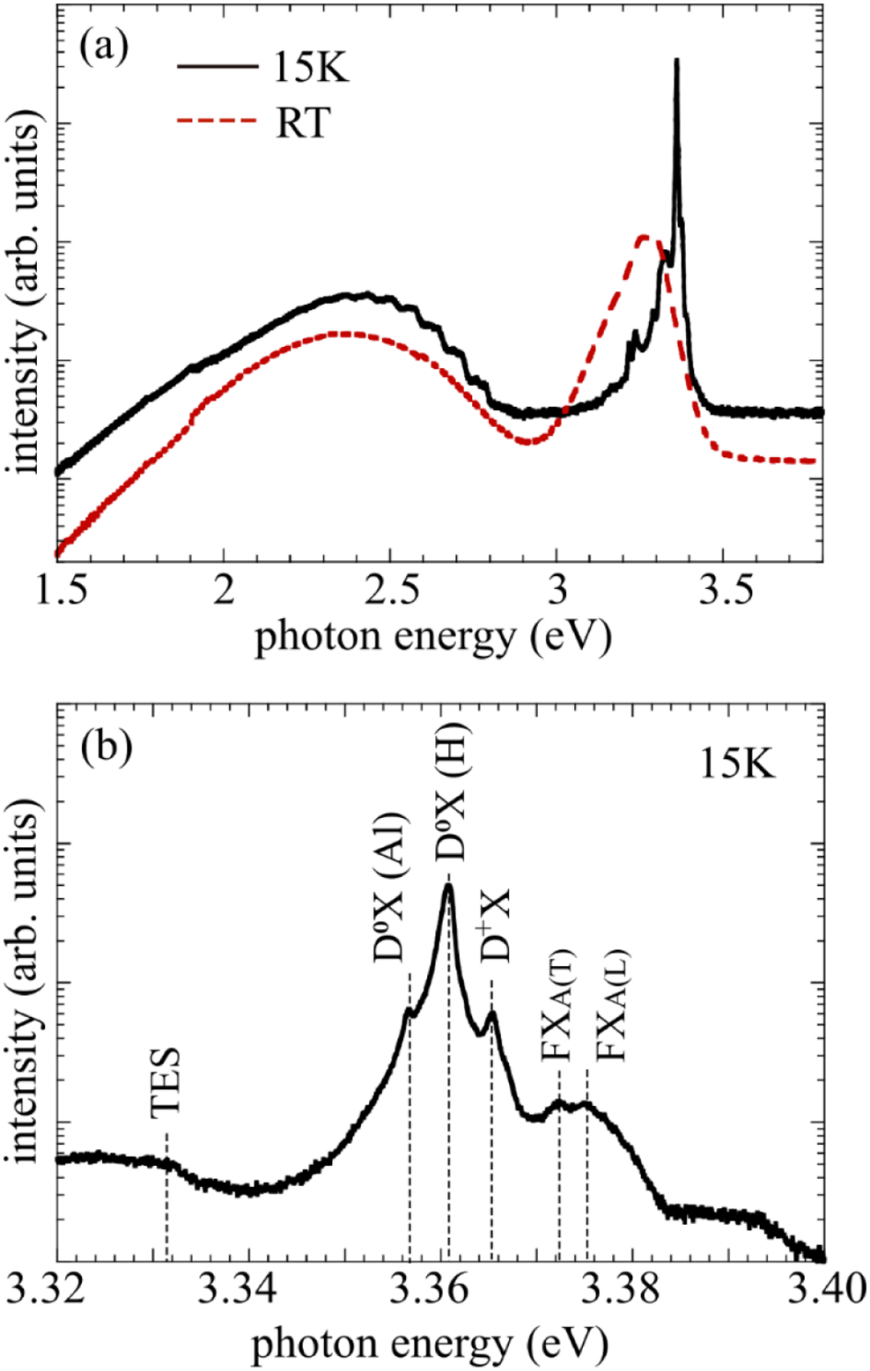
CL spectra of a 500 nm-thick ZnO film on a 1.5 nm-thick MgO buffer layer plotted on a logarithmic intensity scale. (a) CL spectra measured at 15 K and RT. (b) High-resolution CL spectrum measured at 15 K, focusing on the NBE emission region.

The peak at 3.375 eV is attributed to longitudinal B-excitons bound to neutral Al donors. This assignment is supported by the ∼18 meV energy shift from the reported longitudinal free B-exciton,^46^ which is consistent with the typical localization energy (∼16 meV) of a B-exciton bound to a neutral Al donor. Additionally, emission bands were observed between 3.376 and 3.396 eV, which originated from inhomogeneously broadened free exciton emissions, including free A-excitons, transverse free B-excitons, and longitudinal free B-excitons, whose centers have been reported at 3.3768, 3.3821, and 3.3932 eV, respectively.^46^ Such broadening can be attributed to exciton scattering caused by impurities and structural inhomogeneities, such as dislocations and residual strains. Nevertheless, the clear resolution of the D^0^ X lines suggests that, although some inhomogeneities are present, the ZnO film deposited on the MgO buffer layer retains satisfactory optical and structural qualities.

## Conclusions

We have demonstrated that ultrathin MgO buffer layers (*d*_MgO_ = 1–2 nm) enable the epitaxial growth of atomically flat ZnO films on c-plane sapphire substrates despite an 18% lattice mismatch. The resulting ZnO films exhibited a narrow fwhm of 21 arcsec in the 0002 XRC and an RMS surface roughness of 0.11 nm. RHEED and AFM analyses suggest that the MgO buffers form 3D islands and are structurally more consistent with metastable wurtzite structure than with rocksalt one. This ultrathin 3D MgO layer effectively relieves the lattice strain at the MgO/sapphire interface while simultaneously providing a nearly lattice-matched template for ZnO. In contrast, when *d*_MgO_ exceeded 2 nm, a structural transition to the thermodynamically stable rocksalt phase occurred, severely degrading the ZnO crystal quality (XRC-fwhm > 1000 arcsec). In addition to these structural phase effects, the growth temperature played an important role: while a higher *T*_ZnO_ improved the crystal quality as expected, a lower *T*_MgO_ also enhanced the film quality by reducing the *D*/*F* ratio of the adatoms, promoting the formation of smaller 3D islands that further facilitated strain relaxation. Low-temperature CL spectra exhibited clear resolutions of the D^0^ X emission lines, implying the satisfactory optical and structural quality of the films, even in the presence of shallow-donor states. These findings demonstrate that ultrathin MgO layers function as highly effective strain-relief buffers and may pave the way for high-quality epitaxy of other wurtzite-structured semiconductors on lattice-mismatched substrates, offering a promising route for future material integration in advanced electronic and optoelectronic devices.

## Data Availability

The data supporting the findings of this study are available within the article. Additional data are available from the corresponding author upon reasonable request.
